# Editorial: The interaction of NKG2D and its ligands in health and diseases

**DOI:** 10.3389/fimmu.2022.1099580

**Published:** 2022-12-08

**Authors:** Silvana Gaudieri, Hugh T. Reyburn, Mar Vales-Gomez, Chanvit Leelayuwat

**Affiliations:** ^1^ School of Human Sciences, University of Western Australia, Crawley, WA, Australia; ^2^ Division of Infectious Diseases, Department of Medicine, Vanderbilt University Medical Center, Nashville, TN, United States; ^3^ Institute for Immunology and Infectious Diseases, Murdoch University, Murdoch, WA, Australia; ^4^ National Center for Biotechnology, Spanish National Research Council, Madrid, Spain; ^5^ Department of Clinical Immunology and Transfusion Sciences and The Centre for Research and Development of Medical Diagnostic Laboratories, Faculty of Associated Medical Sciences, Khon Kaen University, Khon Kaen, Thailand

**Keywords:** NK cells, NKG2D, MIC - major histocompatibility complex (MHC) class I-related chain, ULBP - UL16 binding protein, disease

The Natural Killer group 2 member D (NKG2D) receptor is expressed on cytotoxic immune cells (NK and specific T cell subsets) and the interaction with its cognate ligands (NKG2DL) leads to immune activation, a process that underpins the host’s immune surveillance of cellular stress and transformation. Not surprisingly, numerous studies, including articles in this section, have examined the role of NKG2D and NKG2DL in viral infections, autoimmune diseases and tumour progression.

The NKG2DL consist of the multicopy major histocompatibility complex (MHC) Class I-related chain (MIC) and UL16 binding protein (ULBP) gene families. As illustrated in this section, several of these members exhibit variation in extracellular domains that impact the interaction with NKG2D and/or variations in other domains that affect presentation on the cell surface and anchorage to the cell membrane (1). Interestingly, there is also a null MICA haplotype in humans with no obvious detrimental phenotype, suggesting compensatory mechanisms within and between the NKG2DL families (2). The observed variation in the receptor/ligand molecules of the NKG2D/NKG2DL axis likely reflects a complex hierarchical system of interactions that is still not completely understood, but mirrors another highly variable system involving the related human leucocyte antigen (HLA) class I molecules and the inhibitory and activating killer immunoglobulin-like receptors (KIRs) (3) on the same target/effector cells. Such complexity in these systems have likely arisen *via* co-evolution with pathogens over-time.

## The NKG2D/NKG2DL axis and viral infections

One aspect of this co-evolution between host and pathogen is the selective pressure exerted by the host’s immune response on the pathogen. As such, we can better understand our own immune system by examining the various immune evasion mechanisms adopted by pathogens (e.g. viruses) to enable survival. These viral adaptations highlight critical components of our immune response as well as provide clues to unravel complex immune systems - learning immunology from viruses! Several pathogens have developed mechanisms to avoid or manipulate the NKG2D/NKG2DL axis using different approaches including increased proteolytic shedding of NKG2DL from the cell membrane resulting in the downregulation of NKG2D on NK cells (and potentially leading to NK cell exhaustion), increased microRNA degradation of transcripts and protein level modifications. In this section, Chaouat et al. use an elegant set of experiments to identify novel immunoevasins of the NKG2D/NKG2DL axis in HHV-6A, a herpesviruses that can be pathogenic in immunocompromised adults. Interestingly, the immunoevasins encoded within the HHV-6A genome selectively downregulate protein levels of only some NKG2DL members (akin to how HIV downregulates select HLA loci), reinforcing the likely differing functions for the closely related members of the multicopy MIC and ULBP gene families.

## The NKG2D/NKG2DL axis and cancer

Both virally infected cells and tumour cells are mainly eliminated by NK cells and cytotoxic T cells. As expected, immune evasion mechanisms utilised by viruses can be similar to those utilised by cancer cells for survival. Given the diversity of the tumour microenvironments and different tumour immune evasion mechanisms, further studies are needed to better understand the role of the NKG2D/NKG2DL axis in the risk of development of different tumours and tumour progression. In this issue, Toledo-Stuardo et al. examined the association of MICA variants and gastric cancer outcomes and identified significant differences in allele frequencies between subjects with gastric adenocarcinoma and normal healthy controls. The association appeared to be mainly driven by a variation in the extracellular region of the MICA protein that likely impacts the structural confirmation of the protein. Examination of this tumour type is particularly interesting as the gastric mucosa is one of the select sites in which there is low level constitutive expression of MICA. The role of MICA in this region in healthy individuals is unclear.

Machuldova et al. provide an extensive review of the role of the NKG2D/NKG2DL axis in acute myeloid leukemia (AML) and its treatment, haematopoietic stem cell transplantation (HSCT). Downregulation of the NKG2D/NKG2DL axis *via* lower expression of NKG2D and increased shedding of NKG2DL resulting in high levels of soluble NKG2DL are associated with poor AML outcome. In the context of HSCT, NK cells have a role in both the graft versus leukemia response and graft versus host disease (GvHD). In this scenario, it is clear that there needs to be a fine balance between activating and inhibitory NK cell receptor and ligand interactions for both tumour and treatment outcomes.

To reduce GvHD, matching patient and donor HLA molecules is routine but here Machuldova and colleagues suggest that MICA and MICB matching is also important in HSCT outcome. This is an interesting suggestion and is supported by historical studies that aimed to match both HLA and non-HLA regions (such as MICA and MICB) in the MHC for HSCT. This technique, termed “MHC block matching”, utilised genomic characteristics of the MHC [i.e. segmental duplications and linkage disequilibrium (LD)] to provide a “signature” of blocks within the MHC (e.g. beta block contained MICA, MICB, HLA-B and -C). The MHC block matching approach appeared to be more effective than HLA matching alone in predicting outcome following unrelated bone marrow transplantation (4). Further examination of the sequences within the amplicons produced from the beta block showed they likely tagged variation at both the MIC and HLA loci (5). The concordance of earlier studies and recent analyses suggest that matching both HLA and MIC will be beneficial for HSCT outcomes.

## The NKG2D/NKG2DL axis as therapy targets

Current immunotherapy approaches to conquer tumours aim to boost anti-tumour immune responses. As mentioned above, a critical component of this anti-tumour response are the cytotoxic effector NK and T cells. The review by Fuertes et al. lays out our current understanding of the role of NK cells in immuno-oncology for different tumour types. The review highlights the current challenges and advantages of pursuing NK cell-based strategies as a critical component of the fight against cancers and strengthens the view that fostering NK-cell mediated effector functions remains high on the list of priorities to improve current anti-tumour treatments. The provided list of pharmaceutical drugs currently in clinical trials that influence the NKG2D/NKG2DL axis *via* promoting the upregulation of NKG2DL on tumour cells, or inhibiting their shedding is complemented by others that may facilitate the reprogramming of associated cells in the tumour microenvironment such as macrophages. This review aligns well with the perspective provided by Alves et al. that discuss advances in the genome editing field and how this can be used to manipulate expression of the NKG2DL molecules and related pathways. Together these two papers provide an insight into the future opportunities of the NKG2D/NKG2DL axis to improve treatment for cancers and other diseases associated with these molecules.

## Author contributions

SG was the main contributor to the draft manuscript with contributions from others to produce the final version. All authors contributed to the article and approved the submitted version.
